# Electrical impedance detects early stages of bone healing: An in vivo explanatory study of tibial fractures in rabbits

**DOI:** 10.1002/jeo2.12048

**Published:** 2024-06-11

**Authors:** Markus Winther Frost, Maria Tirta, Ole Rahbek, Laura Amalie Rytoft, Ming Ding, Ming Shen, Kirsten Duch, Søren Kold

**Affiliations:** ^1^ Department of Orthopaedics Aalborg University Hospital Aalborg Denmark; ^2^ Department of Orthopaedic Surgery & Traumatology Odense University Hospital Odense Denmark; ^3^ Department of Clinical Research University of Southern Denmark Odense Denmark; ^4^ Department of Electronic Systems Aalborg University Aalborg Denmark; ^5^ Unit of Clinical Biostatistics Aalborg University Hospital Aalborg Denmark

**Keywords:** bone healing, experimental research, impedance

## Abstract

**Purpose:**

Healing after bone fracture is assessed by clinical examination and frequent radiographs, which expose patients to radiation and lack standardisation. This study aimed to explore electrical impedance patterns during bone healing using electrical impedance spectroscopy in 18 rabbits subjected to tibial fracture stabilised with an external fixator.

**Methods:**

Impedance was measured daily across the fracture site at a frequency range of 5 Hz to 1 MHz. Biweekly radiographs were analysed using modified anterior‐posterior (AP) radiographic union score of the tibia (RUST). The animals were divided into three groups with different follow‐up times: 1, 3 and 6 weeks for micro‐computer tomography and mechanical testing.

**Results:**

A decreasing trend in impedance was observed over time for all rabbits at lower frequencies. Impedance closest to 5 Hz showed a statistically significant decrease over time, with greatest decrease occurring during the first 7 postoperative days. At 5 Hz, a statistically significant correlation was found between impedance and the modified AP RUST score and between impedance and bone volume fraction.

**Conclusions:**

This study showed that the electrical impedance can be measured in vivo at a distance from the fracture site with a consistent change in impedance over time and revealed significant correlation between increasing radiographic union score and decreasing impedance.

**Level of Evidence:**

Not applicable.

**Abbreviations**
AP: anterior‐posterior
BV/TV: bone volume/tissue volume
NB: formed callus bone
OB: old host cortical bone
RUST: radiographic union score of the tibia

## BACKGROUND

In the United States alone, an estimated 18 million bone fractures were treated in 2010 [24]. Insufficient bone healing occurs in approximately 5%–10% of fractures [17] and imposes high costs both for the individual patient and for society [6]. Patients are, therefore, closely monitored after a fracture to timely intervene if signs of impaired bone healing occur. Presently, fracture healing is assessed in a clinical setting using radiographs and clinical observation. However, sequential radiographs expose the patient to radiation and the degree of bone healing seen on radiographs lags behind biological stages of healing [2]. In addition, clinical observation lacks standardisation and is fraught with physician subjectivity [13].

It is known that biological tissues have electrical properties that are frequency dependent [9, 22]. Over time, changes in the resistive and capacitive properties occur as the bone heals resulting in change in the electrochemical nature of the tissue [23]. A limited number of studies have applied electrical impedance spectroscopy to in vivo animal bone fracture models [7, 8, 10, 12, 13, 14, 16, 20, 25, 26]. All studies reported a consistently increased impedance over time across a range of frequencies, except one study [7]. Of these, only two in vivo studies measured impedance at low frequencies [8, 27]. The animals chosen for the studies varied between mice and rabbits. Mice have a very different bone composition system compared to humans, making the translation of results to humans difficult [16]. Another factor that varied between studies was the electrode placement. Some studies placed the electrodes within the fracture site [13], which is clinically unsound, while others used the metal pins from the external fixator as electrodes giving way to noise from surrounding tissues [12, 14, 21, 25, 26, 27]. The most recent study of Fukase et al. designed and fabricated a microscale sensor placed directly into the fracture gap intraoperatively for wireless, highly sensitive and repeated in vivo electrical impedance spectroscopy [8].

To further investigate the concept of monitoring bone healing with electrical impedance, we developed an in vivo rabbit model with the following distinctions: (1) sensors were placed at a distance from the fracture site, (2) rabbits with a Haversian bone structure similar to humans were used and (3) daily measurement were obtained at a low‐frequency range of 5 Hz to 1 MHz. The primary aim was to investigate whether a pattern of change in electrical impedance occurred over time as the fracture healing progressed. The secondary aim was to investigate whether the electrical impedance correlated to quantitative scores of bone healing obtained from conventional radiograph, micro‐computed tomography (CT) (µCT) and mechanical bending test.

## METHODS

### Study design

The animals were divided into three groups with different follow‐up time prior to euthanization: 1 week (*n* = 5), 3 weeks (*n* = 6) and 6 weeks (*n* = 7). Animals were included until a minimum of five animals had been successfully followed for each observation time. As this was an exploratory study, no prestudy sample size calculation was performed. Electrical impedance was measured immediately as well as 4 h after skin closure and hereafter daily. Radiographs were taken immediately postoperative, and starting from the seventh postoperative day, bi‐weekly anterior‐posterior (AP) radiographs of the tibia were obtained.

### Experimental animals

Twenty‐four New Zealand White female rabbits with a mean body weight of 3.0 ± 0.2 kg were kept in dual cages pre‐operatively. They were allowed to mobilise freely throughout the study. Prior to surgery, the animals were allowed 3 weeks to acclimatise to their surroundings. Care and well‐being of the animals were carried out in accordance with the European Union (EU) legislation and directives regarding animal research.

The rabbits were premedicated using a subcutaneous injection of 2–5 mg/kg Hypnorm (Fentanyl and Fluanisone). Prior to intubation, 1–2 mg/kg Midazolam and 3–6 mg/kg Propofol were given. During surgery, the animal was kept anaesthetised using a continuous intravenous 1–2 mg/kg Fentanyl infusion in combination with 1.5% Sevoflurane. Prophylactic antibiotics 5–10 mg/kg Baytril was injected intramuscularly preoperatively, and the surgical site was shaved and disinfected with betadine. A 60 mm skin incision was performed on the medial side, 1 cm from the tuberosity of the tibia proceeding distal towards the ankle joint, exposing the periosteum and the bone. The muscle in the lateral and posterior compartment was elevated from the bone, as well as the tendons on the medial side. From the medial side, both cortices of the tibia were drilled with 1.8 mm drill using a guide system through soft tissue windows. A total of four Orthofix pins (M300) were inserted. Two pins were inserted proximal and two pins were inserted distal to the planned fracture site.

**Figure 1:**
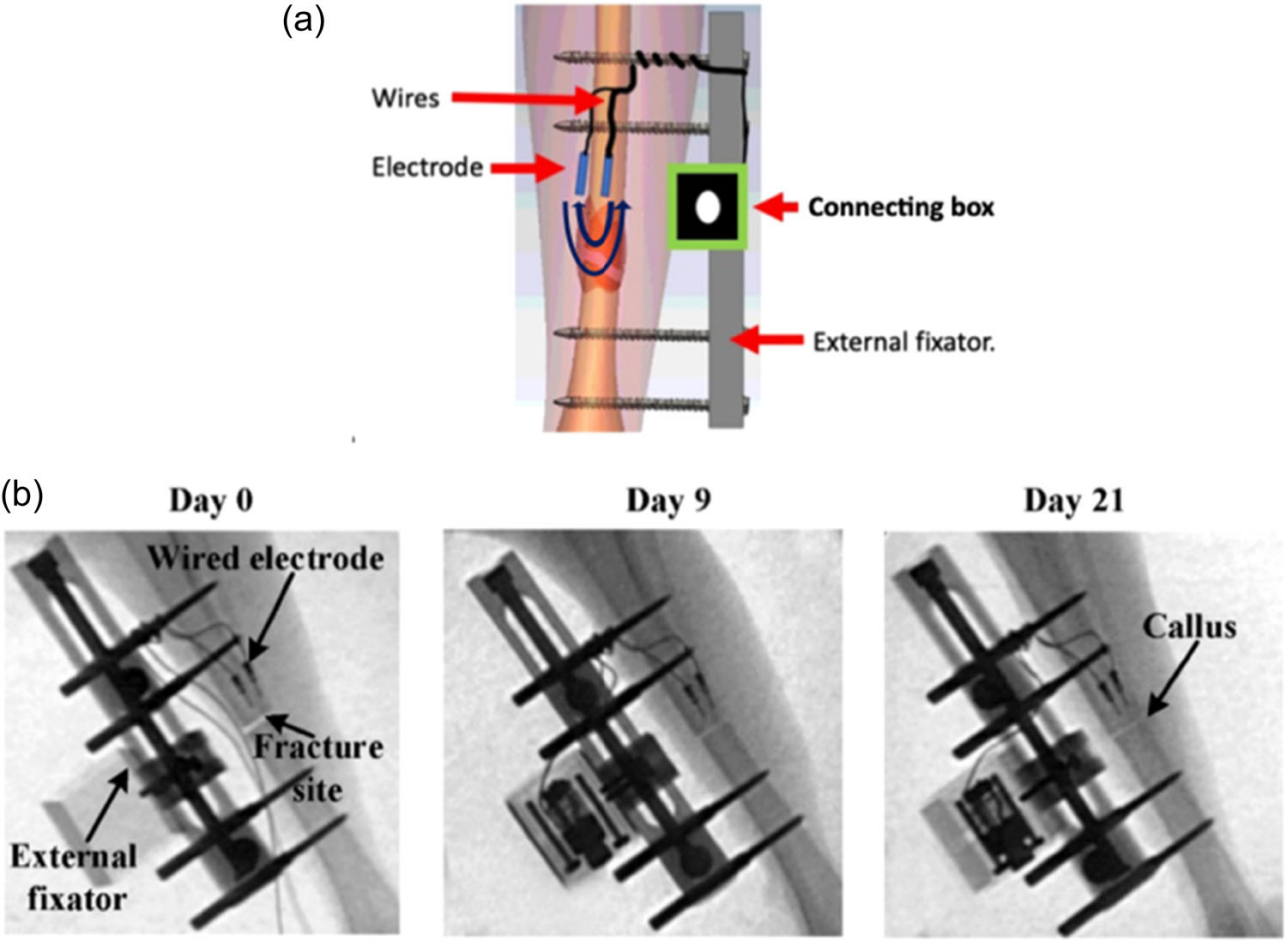
(a) The rabbit tibial fracture model. Two electrodes are positioned proximal to the fracture site, one inside and one outside the medullary cavity on the lateral side and are used to measure electrical impedance transversely at the fracture site. The fracture is stabilised by an external fixator placed medially on the rabbit leg with two pins proximal and two pins distal to the fracture site. A custom‐made plug box that houses the wires from the electrodes is attached to the external fixator (b) X‐rays of a rabbit. X‐rays show that first from Day 21 can radiographic signs (callus) of bone healing be seen.

The fracture was performed as an osteotomy with an oscillating saw at a point equal distal from the proximal to the distal set of pins (Figure 1). In the space between the two proximal Orthofix pins, both cortices of the tibia were drilled and one electrode was introduced into the bone marrow channel. A second electrode was introduced through the hole in the cortex between the proximal pins and placed in the lateral compartment. Both electrodes were located 3 mm proximal to the osteotomy. The wires were tunnelled to the Orthofix pins and later connected to a custom‐made quick plug on the external fixator (Orthofix minirail m100). The muscle fascia and skin were each sutured with resorbable material. Skin glue and spray‐on plaster were applied to help guard against postoperative skin infections. An Orthofix minirail m100 was fixed to the Orthofix pins to stabilise the fracture and the fracture gap was standardised to 1 mm (Figure 1). Postoperative analgesics were administered the first four post‐operative days in the form of subcutaneous 0.01–0.05 mg/kg Temgesic four times a day, in combination with 20 mg/kg Paracetamol two to three times daily.

### Electrical impedance spectroscopy

Impedance was measured using a circuit model consisting of a resistor and capacitator. All electrical impedance measurements were carried out using Digilent Analogue Discovery 2 and Analogue Discovery Impedance Analyzer (Diligent) without using anaesthesia. A frequency range of 5 Hz to 1 MHz was used. Two measurements were conducted at resistance 100 Ω and 1 kΩ, respectively. Numerical measurement data were collected and calculated using a custom script in MATLAB version 9.8 software.

### Radiography and radiographic healing score

Radiographic examination was performed immediately postoperatively. Starting from the seventh postoperative day, bi‐weekly AP radiographs of the tibia were obtained without anaesthesia. Bone healing was scored using the modified AP radiographic union score of the tibia (RUST) score [15]. The medial and lateral cortex were each assigned a number from 1 to 4; 1 = *no callus*, 2 = *callus present*, 3 = *bridging callus* and 4 = *remodelled with no visible fracture line*. Total values for each specimen, therefore, ranged from 2–8 by adding up the scores for the medial and lateral cortices. Lateral radiographs of the anterior and posterior cortices were not performed due to the external fixator occluding the penetration of X‐rays. All X‐ray images were obtained by mobile C‐arm.

### µCT scanning and microarchitectural analysis

At the end of the follow‐up period, the animal was sedated and euthanized with an overdose of Pentobarbital. The tibial bone was harvested and fixated onto a plastic board using fixating pins, followed by removal of the external fixator and sensors. The specimens were stored for 6 weeks in the freezer at −5°C until postmortem testing. µCT scanning was performed using a high‐resolution scanner (µCT 50; Scanco Medical AG) with 90 kV energy, 155 mA intensity and 1000 projections. Ten millimetre length of the tibia was scanned from the fracture centre with proximal and distal lengths of 5 mm each, and the three‐dimensional (3D) reconstruction cubic voxel sizes of µCT images were 10 × 10 × 10 mm^3^. The centre 6 mm were contoured along callus borders to quantify the microarchitectural properties of the newly formed bone in the fracture region [4]. The µCT images were segmented using Scanco software (Scanco, Medical AG) and were divided into newly formed callus bone (NB), old host cortical bone (OB) and a combination of NB and OB [18]. Thus, three image data sets were generated.

For each image, old bone (mainly cortical bone with high density) was differentiated from new callus bone (low density) based on a new algorithm implemented by Scanco (Scanco, Medical AG). Unbiased and assumption‐free 3D methods were used to calculate the microarchitectural properties of the NB and of the OB, and NB + OB as references. Finally, the microarchitectural properties of the NB were reported only. Bone volume fraction (bone volume/tissue volume [BV/TV], %) was the only parameter included in this study.

### Mechanical testing

The samples were thawed at room temperature 2 h before mechanical testing. Three‐point bending tests were performed using an 858 Bionix MTS Hydraulic Material Testing System (MTS System). This test was designed to assess the healing material's ability to resist deformation under load and here we evaluated the bending strength at the fracture healing site [5]. The tibial sample was placed on the two under supports with a distance of 20 mm, and a loading pin applied force at the centre of the fracture site during the test with a displacement rate of 5 mm/min. Load versus displacement data were recorded and converted to stress data to calculate mechanical parameters: maximum stress (MPa), failure energy (kJ/cm^3^) and young modulus (MPa) [3].

### Statistical analysis

Spearman's correlation coefficient was calculated between impedance and modified AP RUST at 16 different frequencies between 5 and 10,000 Hz and bootstrap 95% confidence intervals (CIs) with 1000 bootstrap samples were calculated for relevant measures. Spearman's correlation coefficient was chosen since the modified RUST score and the impedance were not bi‐normal distributed at all frequencies according to Mardia's test. When impedance was not measured at the exact frequency, the closest measurement was chosen.

Of the 16 frequencies, the frequency with the highest absolute correlation coefficient was used to further analyse the impedance data against time. The impedance was evaluated using three linear mixed models with rabbit as random intercept and days since operation as linear fixed effect. The first model was based on Days 0–7 postoperation, the second from Days 8–21 postoperation and the third from Days 22–42 postoperation (based on when evaluation of bone healing process happened). Parameter optimisation was done using restricted maximum likelihood. Residuals were investigated to consider the model fit and was deemed reasonable normal distributed with no clear trend in the residual plot. Variation was illustrated in a figure using least square means for varying days since the operation.

Pearson's correlation coefficients and 95% CI were calculated for the correlation between the impedance measure at the measurement closest to 5 Hz and BV/TV of callus tissue, maximum stress and failure energy.

Significant level was set to 5%. Statistical analysis was performed with the help of a statistician using R version 4.0.0 software. The main statistical packages used were ‘lme4’ and ‘lsmeans’ (see attached code in the Supporting Information for full information). Missing impedance measurements occurred 13 times, these measures were simply omitted from the analysis.

## RESULTS

### Research animals participants

Twenty‐four rabbits were operated. Three rabbits died under intubation. Three rabbits were excluded due to postoperative fracture around the proximal pin sites leading to premature euthanization. The problem with proximal fracture was solved by predrilling to a larger diameter prior to insertion of proximal pins. The remaining 18 rabbits were included in the study and allocated in three groups with different follow‐up time of 1 week (*n* = five rabbits), 3 weeks (*n* = six rabbits) and 6 weeks (*n* = seven rabbits).

### Impedance over time

**Figure 2:**
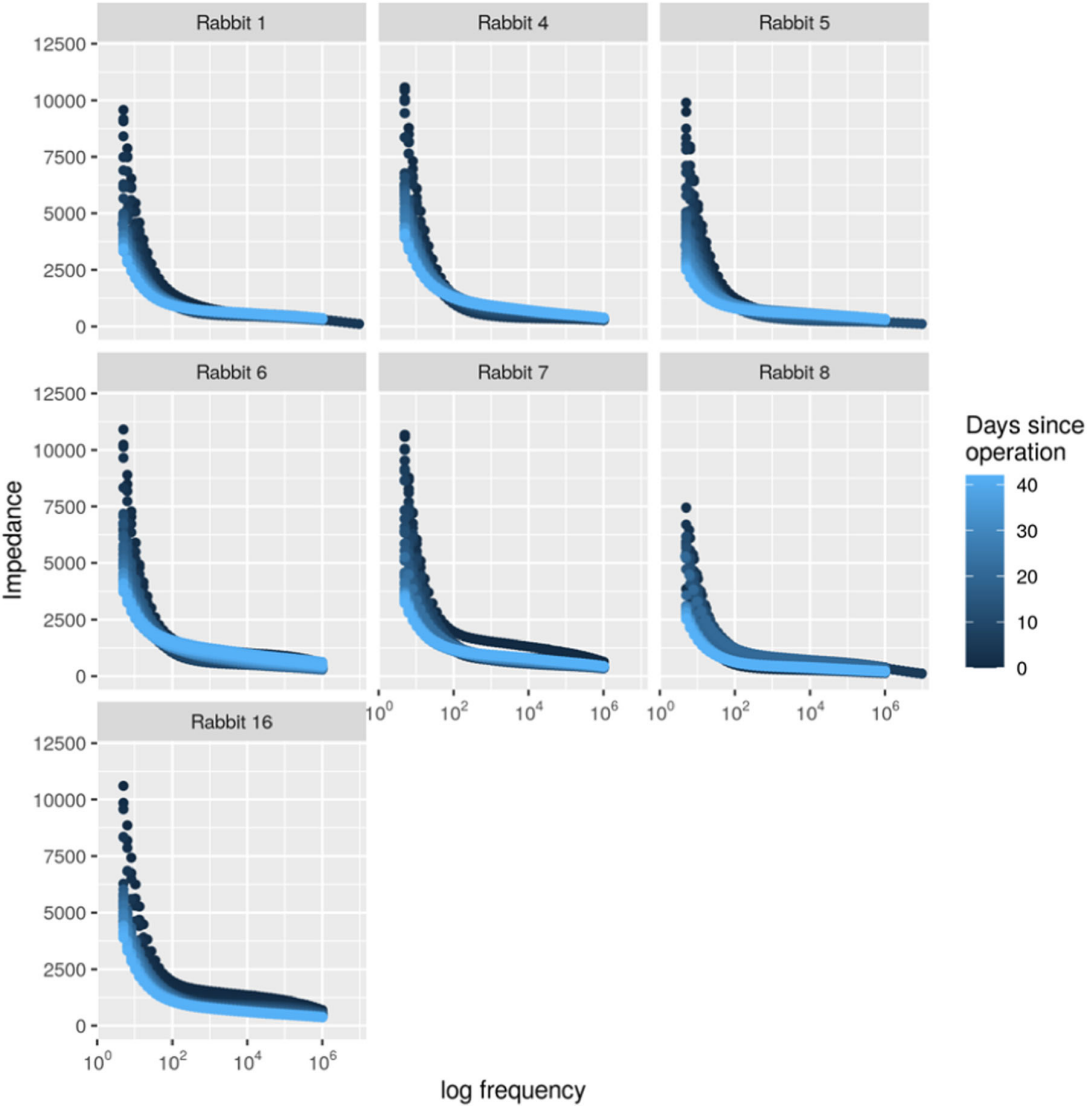
Impedance (Ω) plotted against a range of logarithmic frequencies for the seven rabbits with a 6‐week follow‐up. Each graph represents one rabbit. Impedance was measured for all frequencies each day and each of the 43 days are coloured by a different tone of blue. For all rabbits, the largest spread in impedance was found at the lowest frequency (5 Hz) and at 5 Hz the impedance decreased over time.

At low frequencies, impedance decreased over time for all the rabbits. Figure 2 illustrates the distribution of impedance through the days for the seven rabbits followed for 6 weeks, highlighting the broad range of impedance values over time in low frequencies, contrary to the narrow range of impedance values at higher frequencies. At frequencies above 30 Hz, impedance increased over time from Days 7–42 for some rabbits (Figure 2). At the seventh to tenth postoperative day, the impedance started to change from decreasing to increasing with the point of this reversion being a frequency between 30 and 300 Hz.

**Figure 3:**
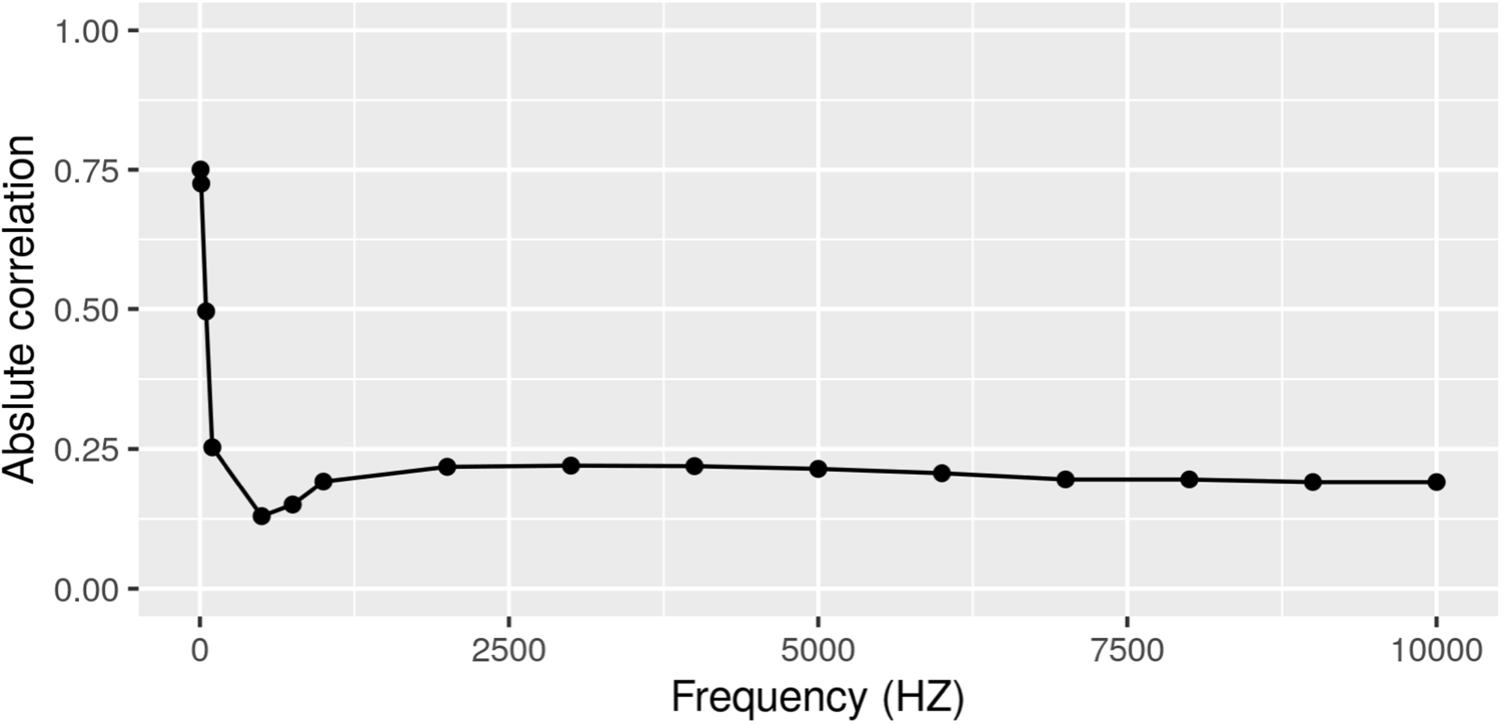
Absolute correlation between modified radiographic union score of the tibia (RUST) scores and impedance measurements. Impedance measurements were at 16 selected frequencies ranging from 5 to 10,000 Hz. The highest absolute correlation between modified anterior‐posterior RUST scores and the impedance measurements was found at the 5 Hz frequency.

**Figure 4:**
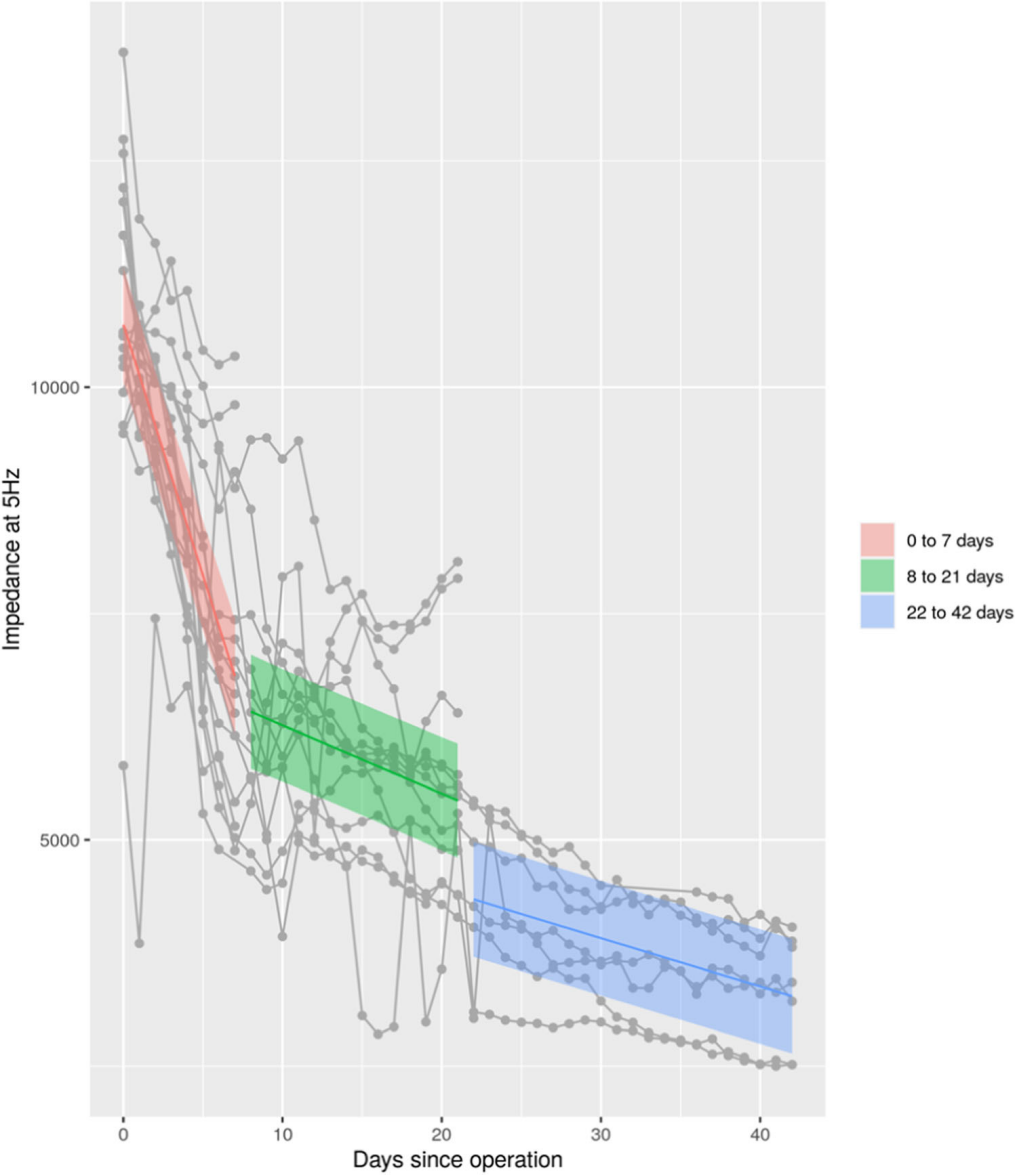
Impedance measured at frequency closest to 5 Hz plotted against days since operation. Measurements for individual rabbits are connected by a line. From Days 0 to 7 measurements were made in 18 rabbits, from Days 8 to 21 in 13 rabbits and from Days 22 to 42 in seven rabbits. Each coloured line represents the average change in impedance between Days 0 and 7 (red line), between Days 8 and 21 (green line) and between Days 22 and 42 (blue line), and also corresponding confidence intervals are shown.

The spread in data between different days was largest at about 5 Hz (Figure 2), suggesting that 5 Hz frequency seems to be more sensitive in detecting changes in the impedance measurement. Additionally, the highest absolute correlation between the modified AP RUST score and the impedance was found at about 5 Hz (Figure 3). Therefore, the impedance was further analysed at an observed frequency closest to 5 Hz. There was an overall trend of decreased impedance over time for all rabbits at the measurement closest to 5 Hz (Figure 4). The rate of decrease was most pronounced the first 7 days postoperatively and the impedance continued to decrease all 6 weeks during healing. Using a linear mixed model for measurements obtained at the frequency of 5 Hz, the average impedance at Day 0 was 10,688 ± 304 Ω. The slope from Days 0 to 7 was estimated as −554 ± 33 Ω/day, the slope for Days 8–21 was estimated to be −75 ± 12 Ω/day and for Days 22–42 the slope was −53 ± 3 Ω/day (Figure 4). This indicates that the impedance decreased rapidly before Day 7 and slower after Day 7.

### Impedance and radiographic healing score

**Figure 5:**
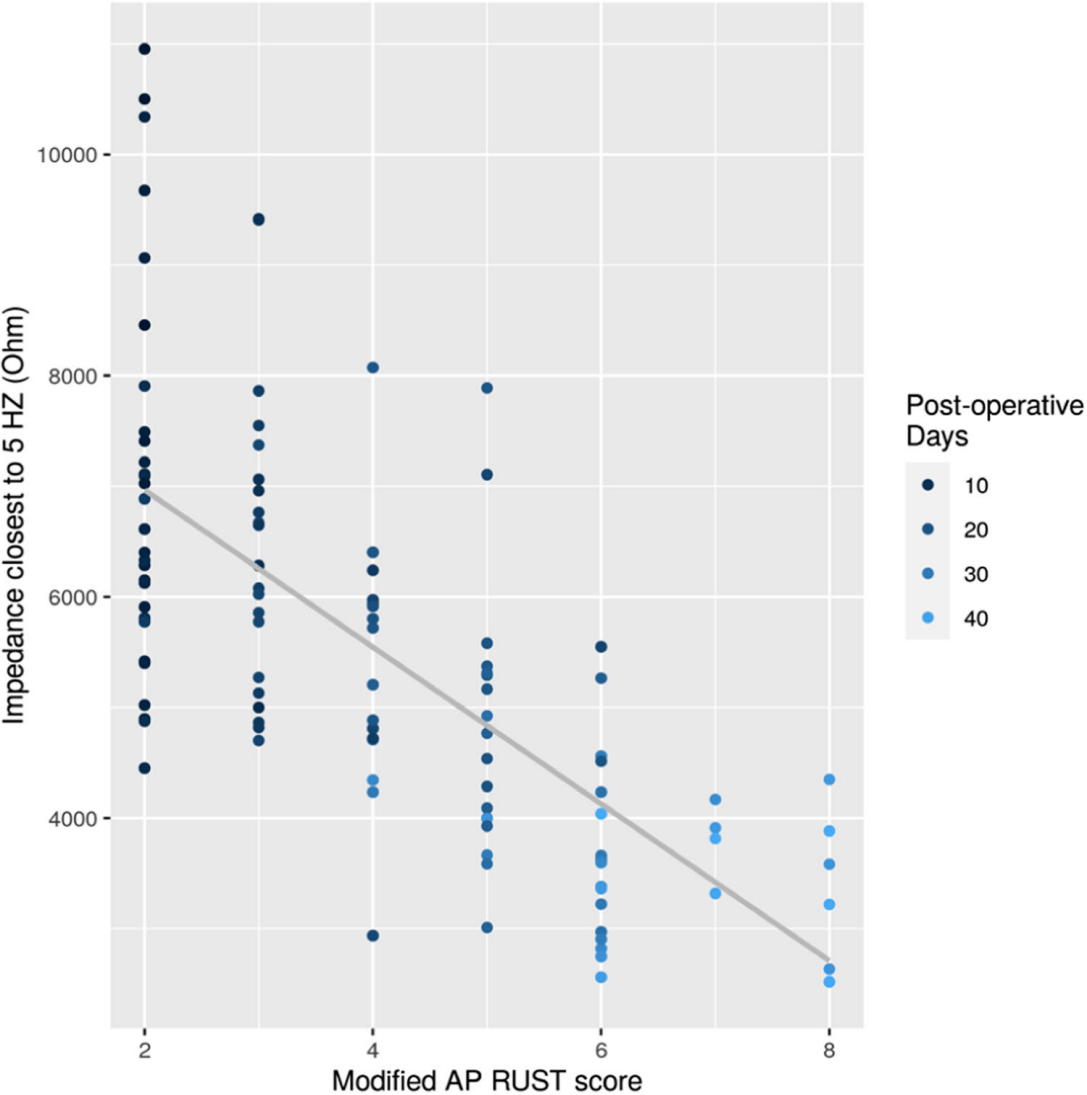
Correlation between impedance measured closest to frequency of 5 Hz and modified anterior‐posterior (AP) radiographic union score of the tibia (RUST) score. Days since the operation was added to illustrate time with the colour turning lighter shades of blue as time passes.

Callus gradually formed around the fracture site, favouring the lateral side. The callus was first visible on radiographs from Week 2 and bridging callus from around Week 4. Spearman's correlation coefficient between impedance and modified AP RUST score at the frequency closest to 5 Hz was −0.75 [95% confidence interval (CI) for the regression coefficient (b) in the context of linear regression analysis: (−0.83; −0.65)]. Figure 5 illustrates the relationship between the modified AP RUST score and the impedance measured at the frequency closet to 5 Hz. The best linear fit was added to the graph to illustrate the tendency. The impedance decreased as the modified AP RUST score increased.

### Postmortem imaging and mechanical testing

**Figure 6:**
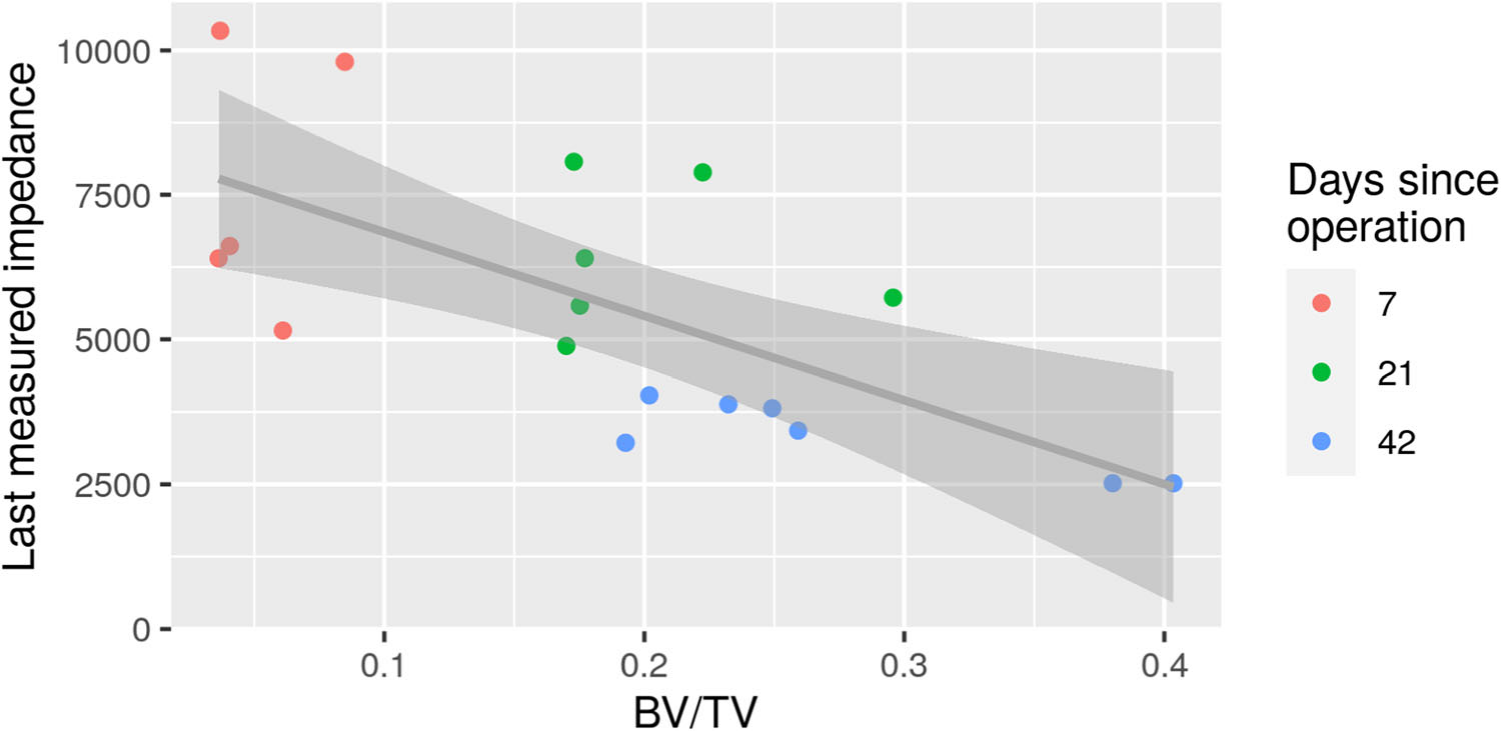
Correlation between last measured impedance at 5 Hz (Ω) frequency immediately prior to euthanization and bone volume/tissue volume (BV/TV) of callus tissue. Euthanization occurred after 1 week for 5 rabbits (red colour), after 3 weeks for 6 rabbits (green colour) and after 6 weeks for 7 rabbits (blue colour).

**Figure 7:**
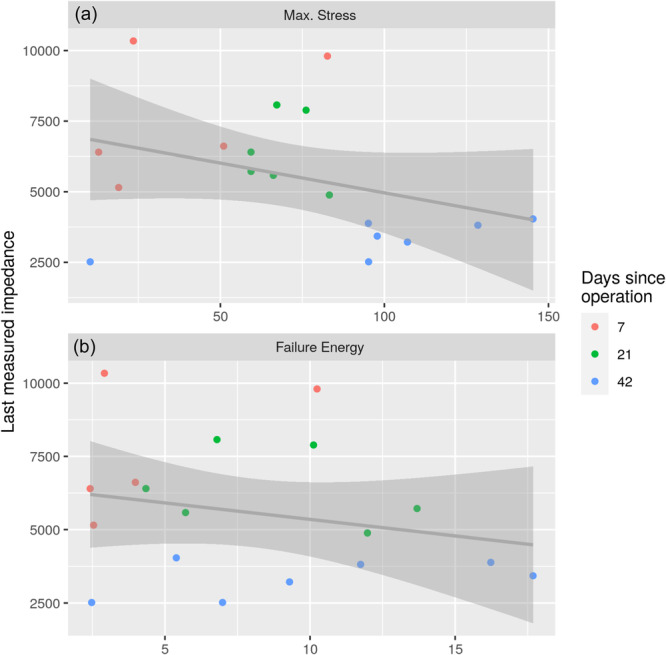
Correlation between last measured impedance at 5 Hz frequency (Ω) immediately prior to euthanization and maximum stress (a), and failure energy (b). Euthanization occurred after 1 week for five rabbits (red colour), after 3 weeks for six rabbits (green colour) and after 6 weeks for seven rabbits (blue colour).

There was a statistically significant correlation between last measured impedance at 5 Hz frequency immediately prior to euthanasia and BV/TV of callus [−0.68, 95% CI: (−0.87; −0.31)] (Figure 6). The best linear fit was added to the graph to illustrate the tendency. The impedance decreased as the BV/TV of callus increased. A statistically significant correlation was also found between last measured impedance at 5 Hz frequency immediately prior to euthanasia and BV/TV of all bone (callus with old bone) [−0.56, 95% CI: (−0.81; −0.13)]. Considering the mechanical testing with three‐point bending, no significant correlation was found between last measured impedance at 5 Hz frequency immediately prior to euthanasia and maximum stress [Figure 7a, correlation = −0.35, 95% CI: (−0.70; 0.14)], failure energy [Figure 7b, correlation = −0.23, 95% CI: (−0.63; 0.26)] or young modulus [−0.28, 95%CI: (−0.66; 0.22)]. Adding the best linear fit to the graphs, the impedance seemed to decrease as the maximum stress or failure energy increased, but not statistically significant (Figure 7).

## DISCUSSION

The study found consistent patterns of change in impedance over time for all rabbits. The largest differences in impedance values over time occurred at the lowest measured frequency of 5 Hz. Analysing the measurements closest to 5 Hz, revealed a clear trend where impedance decreased steeply during the first 7 days followed by a slower decreasing trend.

As impedance measured at 5 Hz decreased over time, the modified AP RUST score measured during biweekly radiographs increased, revealing a statistically significant negative correlation. Previous studies have used radiographs as a way of visually correlating fracture healing to changes in impedance [8, 20, 21, 25, 27]. However, to our knowledge, only two other study utilised a modified RUST score to quantify radiograph results [8, 13], further, Fukase et al. [8] and our study are the only ones to reveal that radiographic changes significantly correlate with impedance in a fracture fixation model. This suggests that impedance spectroscopy has the potential to reduce the number of radiographs needed in postoperative surveillance of bone healing. This is further supported by the finding that impedance measured at 5 Hz also had a significant negative correlation with bone volume fraction detected from µCT of specimens at three different time points. However, the negative correlation between impedance measured at 5 Hz and mechanical parameters (stress, strain) were nonsignificant.

We found changes in impedance measurements varied at different frequencies probably due to impedance being frequency‐dependent for different types of tissue. To our knowledge, only two other studies have measured impedance with a frequency lower than 5 Hz [8, 25]. An in vivo study by Yoshida et al. implanted external nails in fractured rabbit bones and measured impedance at a constant frequency of 2.0 Hz during healing and found that overall impedance increased over time [25]. Furthermore, significant differences were observed between 1 and 2, 3, 4, 5 and 6 weeks (*p* < 0.05) and between 2 and 5 weeks (*p* < 0.05) [25]. Yoshida et al. used externally applied bone pins as electrodes which creates a bone–pin interface that might interfere with the results from the fracture site. In addition, noise from the surrounding soft tissue might influence the signal and therefore provide low specificity for changes in bone healing [25]. Fukase et al. [8] obtained impedance measurements after the 10th postoperative day from microscale sensors placed directly into the fracture gap in rabbits. At 2 kHz an impedance increased over time correlating with radiographic union scores [8]. These findings are in accordance with our results as we found that impedance after the seventh postoperative day increased at higher frequencies between 30 and 300 Hz. However, there are some differences between these two studies, with the major one being that Fukase et al. placed the sensors in the fracture gap while our model applied sensors at a distance from the fracture gap [8]. Our results are also in agreement with published results from other in vivo animal impedance studies, reporting increased impedance over time across a range of higher frequencies, such as 32–90 kHz [10], 20 Hz–1 MHz [13] and 20 Hz–7 kHz [20]. Considering our secondary outcome of µCT, Lin et al. [13] also found a significant correlation between impedance and BV/TV and stated that this finding indicates that impedances correlate to clinically relevant measures of bone healing that would otherwise require expensive µCT evaluation [13].

To our knowledge, the present study is the first to place the electrodes close to, but not within, the fracture gap, with one electrode placed within the bone marrow and the other electrode outside the bone cortex. This allows for impedance measurements over a transverse plane at the fracture site. Lin et al. avoided the use of external pins for obtaining electrical impedance measurements by placing the electrodes within the fracture site [13]. While this reduced the risk of noise from surrounding tissue, it had the disadvantages that the small‐scale sensors were prone to movement, required a connection to external wires to acquire measurements and had low signal‐to‐noise ratios due to the small electrode size, as it is mentioned in their latest study [8]. Although this recent study suggested that wires of a microscale sensor implanted directly into the fracture gap did not interfere with healing progression, in one out of the six rabbits the two wires deviated from the gap at 2 weeks postoperatively, and furthermore no data were provided in one out of the six sensors after implantation [8].

Like Fukase et al. [8], we explored the concept of using impedance for monitoring bone healing in rabbits as rabbits have a cortical bone structure that is more similar to humans than small mammals, such as mice and rats. Thus, the bones of rabbits have the presence of Haversian systems. In contrast, rats and mice do not possess a Haversian system as their bones are too small to permit osteocyte survival without intraosseous circulation. Bone remodelling is facilitated by a Haversian system, and therefore, it might be important to perform studies in animals with Haversian systems in order to translate the findings into that of human bone healing.

In previous studies, it has been common practice for the animal to be anaesthetised when performing the impedance measurements and for this reason, measurements were only obtained a few times a week. The need for anaesthesia to measure impedance is unsound from a clinical perspective. Similarly, the use of biweekly measurements means the risk of electrical fluctuations being missed, especially in the early stages of healing where the bone has yet to mineralise and therefore cannot be seen on radiographs. In our study, daily impedance measurements were performed without the use of anaesthesia. An important finding was that impedance measured at 5 Hz showed a significant and marked decrease over time within the first 7 postoperative days. At this early time callus is often not present on X‐rays (Figure 1b) suggesting that impedance might be able to monitor fracture healing at a much earlier time than conventional X‐rays.

The main limitation of the current study is the lack of non‐healing animals used as a control group. Our impedance equipment has therefore yet to be used to detect stagnant healing. In a study by Lin et al., differences in impedance were demonstrated in a mouse with and without healing [13]. It showed that a mouse with a 0.5 mm bone defect had a significant positive relationship in resistance over time coupled with cartilage and trabecular bone on histology, whereas the mouse with a 2 mm bone defect showed no correlative relationship and an overabundance of fibrous tissue on histology [13].

In all rabbits, the electrode outside the medullary cavity was placed on the lateral cortex resulting in electrical impedance measurements over the lateral side. Further studies are needed to investigate whether multidirectional measurements, by placement of multiple electrodes around the fracture site, must be applied to predict successful bone healing. Electrical impedance was measured in a tibial fracture model, where a transverse fracture was created under highly controlled conditions with a water‐cooled sawblade. However, clinically fractures are sustained under less‐controlled conditions where a spiral fracture often occurs after torsional deformation of the bone.

Additional limitations are the uniform surgical technique and fracture creation, which may not perfectly mimic the variability seen in clinical fractures, limits the generalisability of the results, as well as the fact that optimal frequency for monitoring bone healing in humans may differ from that of rabbits. The 6 weeks postoperation follow‐up might also be a limitation, since it may not encompass the full healing timeline, especially for more severe fractures, while also potential movement of sensors could affect the reliability of impedance measurements. The results from the mechanical test might be influenced by the 6 weeks storage of specimens at dry freezing conditions and µCT scans prior to performing the mechanical tests.

Recent studies have recognised the influence of fibrous tissue around the electrodes on the measured impedance values to be a possible study limitation [13]. They imply that sensor motion could have contributed to the excess fibrous tissue in the fracture gap. In our study, the electrodes were wrapped multiple times around the most proximal pin to prevent movement, but especially the lateral electrode could have been subject to movement during muscle activation of the lower leg. During postmortem bone dissection macroscopic fibrous tissue was visible around the lateral electrode. Postmortem histology is needed if the consequence of fibrous tissue on impedance is to be better understood.

Future studies will work to use electrical impedance spectroscopy in a similar rabbit model with a critical‐sized defect. This will provide information on changes in electrical impedance over time in a model where healing is not expected to occur. Additional studies will work to integrate wireless data transfer to provide constant real‐time data on fracture healing. This has the potential to provide patients with personalised fracture treatment plans and the ability to avoid in‐person follow‐up appointments. Structural changes to currently implanted materials must be made to accommodate the sensors. The sensors might need to be placed at multiple sites to obtain multiaxial measurements over the fracture site in both transverse and longitudinal directions. Further steps also involve the model being tested in in vivo large animal models prior to human clinical trials. Additionally, a recent study by Banerjee et al. developed an analytical method combining electrical impedance spectroscopy and machine learning for the quantitative assessment of bone mineral content [1], which can be another innovational part of electrical impedance spectroscopy use.

In conclusion, this study showed that electrical impedance spectroscopy measurements obtained by electrodes placed at a distance to the fracture site can be used for monitoring fracture healing in a rabbit tibial fracture model. Impedance measured closest to 5 Hz frequency decreased statistically significant over time with the steepest rate of decrease occurring during the first 7 postoperative days. A significant negative correlation was found between impedance measured closest to 5 Hz frequency and modified radiographic union scores. The results were congruent with the study's hypotheses. The study suggests that electrical impedance spectroscopy could follow bone healing and potentially supply the physician with quantitative information about healing.

## AUTHOR CONTRIBUTIONS

Markus Winther Frost, Laura Amalie Rytoft, Maria Tirta, Ming Shen, Kirsten Duch, Ole Rahbek and Søren Kold designed the research study. Markus Winther Frost and Laura Amalie Rytoft performed the rodent surgeries. Markus Winther Frost, Maria Tirta, Laura Amalie Rytoft, Ming Shen, Ming Ding and Kirsten Duch collected and analysed the data and drafted the manuscript, while Ole Rahbek and Søren Kold provided conceptual advice and critically revised the paper. All authors have read and approved the final submitted manuscript.

## CONFLICT OF INTEREST STATEMENT

The authors declare no conflict of interest.

## ETHICS STATEMENT

This study was approved by the Danish Animal Experiments and Inspectorates under the Danish Ministry of Justice (Journal number: 2020‐15‐0201‐00544). All care and well‐being of the animals was carried out in accordance with the EU legislation and directives regarding animal research. The principles of the 3Rs (Replacement, Reduction, Refinement) in animal research were followed. Simulations have been carried out [11]. To reduce the risk of including animals for a nonfunctional model, pilot tests were performed [7]. Minimising pain and distress at the highly specialised animal facility ensured refinement. The study was reported in accordance with Animal Research: Reporting of In Vivo Experiments guidelines 2.0 [19].

## Supporting information

Supporting information.

## Data Availability

The data sets used and/or analysed during the current study are available from the corresponding author on reasonable request.
