# Supplementary material for: Electrical impedance detects early stages of bone healing: An in vivo explanatory study of tibial fractures in rabbits
Source: J Exp Orthop. 2024 Jun 11;11(3):e12048. doi: 10.1002/jeo2.12048 (PMC11165676; doi:10.1002/jeo2.12048)
Supplement: Supplementary file 1 — Supporting information. [file JEO2-11-e12048-s001.pdf]

Supplementary Information: Coding in R

Electrical Impedance Detects Early Stages of Bone Healing. An In Vivo Study of Tibial Fractures in Rabbits.

Code in R

```
# Packages
require(DescTools)
require(data.table)
require(ggplot2)
require(rcompanion)
require(lme4)
library(tidyverse)
library(estimatr)
require(lsmeans)
library(scales)
library(QuantPsyc)

#Include data
data <- setDT(read.csv("Data/to_publish.csv"))

# make relevant frequency cut
freq_cut <- c(c(5,10,50,10^2,500,7.5*10^2),seq(from=1000,to=10000,by=1000))

data[,paste0("freq_cut",freq_cut):=lapply(freq_cut,
                      function(x) Closest(Frequency,x)==Frequency),
     by=.(date,kanin)]
data[,last_measure:=days_since_op==max(days_since_op),by=kanin]

# Figure 2 ----
data[,kanin_dum:=sapply(as.character(kanin),
                      function(x) switch(x,"290"=1,"328"=2, "330"=3, "346"=4,
                                         "356"=5, "399"=6, "402"=7, "408"=8,
                                         "578"=9, "590"=10, "619"=11, "628"=12,
                                         "649"=13, "656"=14, "658"=15, "684"=16,
                                         "686"=17, "693"=18, "703"=19))]

gg1 <- ggplot(data=data[like(kanin,"356|290|408|346|399|402|684")],
              aes(x=Frequency,y=Trace_Z,col=days_since_op))+
  geom_point()+
  scale_x_log10(limits = c(1,1.5e7), expand = c(0, 0),
                breaks = trans_breaks("log10", function(x) 10^x),
                labels = trans_format("log10", math_format(10^.x)))+
  facet_wrap(~kanin_dum)+
  labs(color='Days since \noperation')+
  scale_y_continuous(limits=c(0,12000))+
  ylab("Impedance")+
  xlab("log frequency")
```

```
# Figure 3-----
corr_s <- lapply(freq_cut,
                 function(z) cor.test(x=data[!is.na(Total)&get(paste0("freq_cut",z
))==TRUE,
                                                        Trace_Z],
                                                   y=data[!is.na(Total)&get(pa
ste0("freq_cut",z))==TRUE,
                                                        Total],
                                                   method="spearman"))

ssd <- sapply(freq_cut,
                 function(z) sd(x=data[!is.na(Total)&get(paste0("freq_cut",z))==TR
UE,
                                             Trace_Z]))

mult_test <- lapply(freq_cut,
                 function(z) mult.norm(data[!is.na(Total)&get(paste0("freq_cut",z)
)==TRUE,
                                             .(Trace_Z,Total)])$mult.test)

# correlations
out <- data.table(corr=sapply(corr_s,function(x) x[[4]]),
                  p.value=sapply(corr_s,function(x) x[[3]]),
                  cut=freq_cut)
# spread
out_sp <- data.table(sd=ssd,
                     cut=freq_cut)

# normality
out_nn <- data.table(cut=freq_cut,
                     skew_test=sapply(mult_test, function(x) x[1,3]),
                     kur_test=sapply(mult_test, function(x) x[2,3]))
#Plot of correlation
gg <- ggplot(data=out,aes(x=cut,y=abs(corr)))+geom_line()+
  geom_point()+
  ylim(c(0,1))+ylab("Absolute correlation")+
  xlab("Frequency (HZ)")

# Bootstrap CI for 5 frequency
spearmanRho(x=data[!is.na(Total)&freq_cut5==TRUE,Trace_Z],
            y=data[!is.na(Total)&freq_cut5==TRUE,Total],
            method="spearman",R=1000,ci=TRUE)

# Figure 4--------
dt <- data[freq_cut5==TRUE,]
```

```r
#create 3 linear models
fit7 <- lmer(Trace_Z~days_since_op+(1|kanin),
             data=dt[days_since_op<=7],REML=TRUE)
fit21 <- lmer(Trace_Z~days_since_op+(1|kanin),
              data=dt[days_since_op>7&days_since_op<=21],REML=TRUE)
fit42 <- lmer(Trace_Z~days_since_op+(1|kanin),
              data=dt[days_since_op>21],REML=TRUE)

# Model summary
coef(summary(fit7))
confint.merMod(fit7,method="Wald")
coef(summary(fit21))
confint.merMod(fit21,method="Wald")
coef(summary(fit42))
confint.merMod(fit42,method="Wald")

# calculate least sqr confidence intervals.
ls7<- summary(lsmeans(fit7,~1,by="days_since_op",
                      at=list(days_since_op=0:7)))

 ls21<- summary(lsmeans(fit21,~1,by="days_since_op",
                        at=list(days_since_op=8:21)))

ls42<- summary(lsmeans(fit42,~1,by="days_since_op",
                        at=list(days_since_op=22:42)))

dd <- data.table(mm=c(ls7$lsmean,ls21$lsmean,ls42$lsmean),
                 upr=c(ls7$upper.CL,ls21$upper.CL,ls42$upper.CL),
                 lwr=c(ls7$lower.CL,ls21$lower.CL,ls42$lower.CL),
                 days_since_op=0:42,
                 model=c(rep("0 to 7 days",8),
                         rep("8 to 21 days",14),
                         rep("22 to 42 days",21)))
dd[,model:=factor(model,levels=c("0 to 7 days",
                                "8 to 21 days",
                                "22 to 42 days"))]
# Plot the models
g <- ggplot(aes(x=days_since_op,y=Trace_Z,group=kanin),
            data=dt)+
  geom_line(col="darkgrey")+
  geom_point(col="darkgray")+
  xlab("Days since operation")+
  ylab("Impedance at 5Hz")+
  geom_line(aes(x=days_since_op,group=NULL,y=mm,
                col=model),data=dd)+
  geom_ribbon(aes(ymin=lwr,ymax=upr,x=days_since_op,group=NULL,y=mm,
                  col=NULL,fill=model),
              data=dd,alpha=0.4)+
```

```
  guides(col=guide_legend(title=" "))+
  guides(col="none",fill=guide_legend(title=""))


# Figure 5 ------
fig5 <- ggplot(aes(x=Total,y=Trace_Z,col=days_since_op),
               data=data[freq_cut5==TRUE&!is.na(Total)])+
  geom_point()+
  geom_smooth(method="lm",se=FALSE,col="grey")+
  guides(col=guide_legend(title="Post-operative\nDays"))+
  xlab("Modified AP RUST score")+
  ylab("Impedance closest to 5 HZ (Ohm)")

# Figure 6 ------
gg_61 <- ggplot(aes(x=as.numeric(Callus_bvtv),y=Trace_Z,
                    col=factor(days_since_op)),
                data=data[freq_cut5==TRUE&last_measure==TRUE])+
  geom_point()+geom_smooth(aes(col=NULL),method="lm",formula= y~x,col="darkgray")+
  ylab("Last measured impedance")+
  xlab("BV/TV")+
  guides(col=guide_legend(title="Days since \noperation"))

p.cor_61 <- cor.test(data[freq_cut5==TRUE&last_measure==TRUE,as.numeric(Callus_bvt
v)],
                     data[freq_cut5==TRUE&last_measure==TRUE,Trace_Z],
                     method="pearson")

p.cor_62 <- cor.test(data[freq_cut5==TRUE&last_measure==TRUE,as.numeric(TotalBone_
bvtv)],
                     data[freq_cut5==TRUE&last_measure==TRUE,Trace_Z],
                     method="pearson")
# Figure 7 ------
dt <- data[freq_cut5==TRUE&last_measure==TRUE,
           .(kanin,Trace_Z,`max..stress`,Failure.Energy,days_since_op)]
setnames(dt,new=c("A. Max. Stress","B. Failure Energy"),
               old=c("max..stress","Failure.Energy"))

dt.long <- melt(dt,id.vars=c("kanin","Trace_Z","days_since_op"),
                measure.vars=c("A. Max. Stress","B. Failure Energy"))

gg_7 <- ggplot(aes(x=value,y=Trace_Z, col=factor(days_since_op)),
               data=dt.long)+
  geom_point()+
  geom_smooth(aes(col=NULL),method="lm",formula= y~x,col="darkgray")+
  ylab("Last measured impedance")+
  xlab("")+
  guides(col=guide_legend(title="Days since \noperation"))+
  facet_wrap(~variable,scale="free",nrow = 2,shrink=FALSE)
```

```
p.cor_71 <- cor.test(data[freq_cut5==TRUE&last_measure==TRUE,as.numeric(max..stres
s)],
                     data[freq_cut5==TRUE&last_measure==TRUE,Trace_Z],
                     method="pearson")
p.cor_72 <- cor.test(data[freq_cut5==TRUE&last_measure==TRUE,as.numeric(Failure.En
ergy)],
                     data[freq_cut5==TRUE&last_measure==TRUE,Trace_Z],
                     method="pearson")
p.cor_73 <- cor.test(data[freq_cut5==TRUE&last_measure==TRUE,as.numeric(max..E.mod
)],
                     data[freq_cut5==TRUE&last_measure==TRUE,Trace_Z],
                     method="pearson")
```
